# Interaction of human HelQ with DNA polymerase delta halts DNA synthesis and stimulates DNA single-strand annealing

**DOI:** 10.1093/nar/gkad032

**Published:** 2023-01-31

**Authors:** Liu He, Rebecca Lever, Andrew Cubbon, Muhammad Tehseen, Tabitha Jenkins, Alice O Nottingham, Anya Horton, Hannah Betts, Martin Fisher, Samir M Hamdan, Panos Soultanas, Edward L Bolt

**Affiliations:** School of Life Sciences, University of Nottingham, Nottingham, UK; School of Life Sciences, University of Nottingham, Nottingham, UK; School of Life Sciences, University of Nottingham, Nottingham, UK; Bioscience Program, Biological and Environmental Science and Engineering, King Abdullah University of Science and Technology (KAUST), Thuwal, Saudi Arabia; School of Life Sciences, University of Nottingham, Nottingham, UK; School of Life Sciences, University of Nottingham, Nottingham, UK; School of Life Sciences, University of Nottingham, Nottingham, UK; Biodiscovery Institute, School of Chemistry, University of Nottingham, Nottingham, UK; Nanna Therapeutics, Cambridge, UK; Bioscience Program, Biological and Environmental Science and Engineering, King Abdullah University of Science and Technology (KAUST), Thuwal, Saudi Arabia; Biodiscovery Institute, School of Chemistry, University of Nottingham, Nottingham, UK; School of Life Sciences, University of Nottingham, Nottingham, UK

## Abstract

DNA strand breaks are repaired by DNA synthesis from an exposed DNA end paired with a homologous DNA template. DNA polymerase delta (Pol δ) catalyses DNA synthesis in multiple eukaryotic DNA break repair pathways but triggers genome instability unless its activity is restrained. We show that human HelQ halts DNA synthesis by isolated Pol δ and Pol δ-PCNA-RPA holoenzyme. Using novel HelQ mutant proteins we identify that inhibition of Pol δ is independent of DNA binding, and maps to a 70 amino acid intrinsically disordered region of HelQ. Pol δ and its POLD3 subunit robustly stimulated DNA single-strand annealing by HelQ, and POLD3 and HelQ interact physically *via* the intrinsically disordered HelQ region. This data, and inability of HelQ to inhibit DNA synthesis by the POLD1 catalytic subunit of Pol δ, reveal a mechanism for limiting DNA synthesis and promoting DNA strand annealing during human DNA break repair, which centres on POLD3.

## INTRODUCTION

DNA strand breaks are repaired by homologous recombination (HR) and micro-homology mediated end-joining (MMEJ) processes that are reliant on new DNA synthesis. DNA polymerases synthesise DNA in these contexts by extending D-loops generated by a recombinase Rad51/RecA/RadA, or from base-paired DNA microhomologies (1–8). Eukaryotic DNA polymerase delta (Pol δ) participates in multiple modes of DNA break repair by HR (2,4,7,9) that each recover cells from collapsed or blocked DNA replication and transcription, but may also trigger mutagenesis, according to their context and the extent of DNA synthesis. In DNA break repair by break-induced replication (BIR) or microhomology-mediated BIR (MM-BIR) DNA replication triggers genetic rearrangements, tandem duplications and mutagenesis characteristic of cells coping with chronic DNA damage and replication stress (4,10–13).

Mechanisms that limit or prevent mutagenic DNA synthesis during HR include deployment of helicase enzymes that are conserved from yeasts to humans to prevent or dissociate D-loop DNA structures, antagonising the priming of new DNA synthesis from a strand break (14–20). Helicases can achieve this by ATP-dependent translocation of DNA at D-loops, which disrupts base-pairing between the replication priming DNA strand and its template, and may also displace recombination enzymes from DNA, reviewed in (21). The DNA helicase HelQ hydrolyses ATP/dATP, but not other trinucleotides, when it is bound to single-strand DNA (22), powering its translocation as a dimer with 3’ to 5’ directionality (22,23). It contributes to DNA break repair in metazoans but is absent from yeasts. Loss of HelQ (*HELQ^−^^/^^−^*) in cells predisposes to cancers and infertility (24–27) and corresponds to increased frequencies of tandem DNA duplications and long-tracts of DNA repair synthesis during homologous recombination (28,29) and reduced repair by synthesis-dependent strand annealing (SDSA) (30). HelQ also promotes DNA strand annealing in contexts distinct from MMEJ-like DNA repair by its homologue PolQ, a polymerase-helicase that is also found only in metazoans (28). Opposing DNA unwinding and annealing activities of HelQ may be balanced through its interaction with RPA that stimulates its DNA strand annealing function (23,29), and with Rad51 that stimulates its helicase activity (29). However, it is not clear how HelQ may limit DNA replication to shorter tracts that prevent mutagenesis in the form of DNA duplications, as indicated from genetics. Here we show that human HelQ halts DNA synthesis by direct targeting of Pol δ, and that this stimulates HelQ to anneal homologous single-stranded DNA. Human Pol δ is a complex comprising four subunits (POLD1-4), and we further show that HelQ physically and functionally interacts with POLD3 but not with POLD1, D2 or D4. The data identify a plausible mechanism in which HelQ restrains DNA synthesis during homologous recombination to protect against genome instability in humans and other metazoans.

## MATERIALS AND METHODS

### Protein purification

Human HelQ and HelQ^ΔWHD^ proteins were purified as described in (23). The N-HelQ fragment of HelQ (amino acids 1–241) (23) was expressed from *Escherichia coli* codon optimised DNA (GeneArt, Life Technologies) cloned into pET14b (NcoI and Hind III) providing an N-terminal (His)_6_ affinity tag. The resulting plasmid (pTJ09) was used to generate mutant N-HelQ proteins, except for N-HelQ^ΔRPAi^ that was also synthesised in GeneArt and cloned in to pET14b. N-HelQ proteins were expressed in BL21 A.I. cells grown in ampicillin LB broth at 37°C with shaking, by adding IPTG (0.5 mM) and L-arabinose (0.2% w/v) at OD_600_ 0.6 for 3 h. Cells re-suspended in buffer A (20 mM Tris–HCl pH 7.5, 1 M NaCl, 20 mM imidazole and 5% glycerol) were lysed by sonication and the soluble protein supernatant was loaded onto a pre-charged HisTrap HP 5 ml affinity column (Cytiva) pre-equilibrated with buffer A. Bound proteins were eluted in a linear gradient of 20–500 mM Imidazole, and fractions containing N-HelQ were dialyzed into buffer at 4°C (20 mM Tris–HCl pH 7.5, 50 mM NaCl and 5% glycerol) for loading onto pre-equilibrated 1 ml HiTrap Q HP (Cytiva), and bound proteins were eluted in buffer QB (20 mM Tris–HCl pH 7.5, 1 M NaCl and 5% glycerol). Fractions containing N-HelQ were loaded on to a HiLoad 16/600 Superdex 200 pg column (GE Healthcare) in 20 mM Tris–HCl pH 7.5, 100 mM NaCl, 5% glycerol, collecting N-HelQ fractions that were concentrated in a 1 ml HiTrap Q HP column as previously, for dialysis into (20 mM Tris–HCl pH 7.5, 200 mM NaCl, and 20% glycerol) and storage at –80°C.

Purification of human DNA polymerase δ complex (PolD1-4) is described in (31), and DNA polymerase κ (PolK) in (32). Human DNA polymerase η (PolH) was expressed in *E. coli* strain BL21 (DE3) with an N-terminal (His)_6_-SUMO tag from a pE-SUMO-pro expression vector (LifeSensors). Cells grown in 2YT media at 24°C to an OD_600_ of 0.8 and then induced with 0.1 mM isopropyl β-d-thiogalactopyranoside (IPTG) and incubated for 19 h at 16°C. Cells collected by centrifugation were re-suspended in lysis buffer (50 mM Tris–HCl pH 8.0, 750 mM NaCl, 40 mM imidazole, 5 mM β-mercaptoethanol, 0.2% NP-40, 1 mM PMSF, 5% glycerol and EDTA free protease inhibitor cocktail tablet/50ml (Roche, UK)). All further steps were performed at 4°C. Cells were lysed by adding 2 mg/ml lysozyme and sonication. Cell debris was removed by centrifugation (22 040 *g*, 60 min) and supernatant loaded onto HisTrap HP 5 ml affinity column (Cytiva) pre-equilibrated with buffer A (50 mM Tris–HCl pH 7.5, 500 mM NaCl, 40 mM imidazole, 5 mM β-mercaptoethanol and 5% glycerol). Bound proteins eluted in a linear gradient of buffer B (50 mM Tris–HCl pH 7.5, 500 mM NaCl, 500 mM imidazole, 5mM β-mercaptoethanol and 5% glycerol). The peak fractions of polymerase η were pooled and dialyzed overnight in buffer (50 mM Tris–HCl pH 7.5, 500 mM NaCl, 5 mM β-mercaptoethanol and 5% glycerol) in the presence of SUMO protease (LifeSensors) to remove the SUMO tag to generate native polymerase η. The dialyzed sample was re-loaded onto HisTrap HP 5ml using buffers A and B and un-tagged polymerase η was collected in flow-through fractions. These were concentrated and loaded onto HiLoad 16/600 Superdex 200 pg (Cytiva) equilibrated with storage buffer (50 mM Tris–HCl pH 7.5, 300 mM NaCl, 10% glycerol and 1 mM DTT). Fractions containing polymerase η were concentrated, flash-frozen and stored at –80°C.

The human Pol δ sub-units POLD1, POLD2, POLD3 and POLD4 were also expressed and purified individually in *E. coli* BL21 A.I., each with an N-terminal (His)_6_ tag from plasmids generated by GeneArt (ThermoFisher Scientific). For each, cells were grown in LB broth at 37°C until OD_600_ of 0.6 when IPTG (0.5 mM) and L-arabinose (0.2% w/v) were added. Growth was continued for 18 h at 20°C before harvesting cells and resuspending in 50 mM Tris–HCl pH 8.0, 1 M NaCl, 5% glycerol containing phenylmethylsulphonyl fluoride (PMSF) (0.5 mM). Cells were lysed by sonication, and soluble proteins recovered by centrifugation. To purify POLD1 and POLD3, cell lysate was passed into a 5 ml Ni-NTA column (GE Healthcare) pre-equilibrated with buffer A (50 mM Tris–HCl pH 8.0, 40 mM imidazole, 1 M NaCl, 5% glycerol). Bound protein was eluted using buffer A containing extra 500 mM imidazole. Fractions containing POLD1/POLD3 protein were collected and concentrated to 4 ml using VivaSpin centrifugal concentrator (Sartorius) and loaded onto a HiLoad 16/600 Superdex 200 pg column (GE Healthcare) pre-equilibrated with 50 mM Tris–HCl pH 8.0, 100 mM NaCl, 5% glycerol. Eluted fractions containing target proteins were then passed into a 1 ml heparin column (GE Healthcare) pre-equilibrated using buffer HA (50 mM Tris–HCl pH 8.0, 50 mM NaCl and 5% glycerol). Both POLD1 and POLD3 bound to heparin, eluted in buffer HB (50 mM Tris–HCl pH 8.0, 1 M NaCl and 5% glycerol). Concentrated protein was dialysed into Tris–HCl pH 8.0, 200 mM NaCl and 20% glycerol for storage at –80°C.

POLD2 was purified using Ni-NTA column and HiLoad 16/600 Superdex 200 pg column as described previously (in POLD1 and POLD3 purification). Because POLD2 does not bind to heparin column, so fractions eluted from HiLoad 16/600 Superdex 200 pg column were load into a 1 ml HiTrap Q HP column pre-equilibrated using buffer HA. Bound proteins were directly eluted from the column using buffer HB. The concentrated PolD2 was dialysed for storage as described previously (in POLD1 and POLD3 purification).

POLD4 was pure to apparent homogeneity after a Ni-NTA column and was dialysed and stored as for the other sub-units. We generated and purified human RPA protein as described in (23), and human PCNA as described in (31). *E. coli* DNA polymerase I was purchased from New England Biolabs. *E. coli* DNA polymerase III and DnaE were from Dr Michelle Hawkins, University of York, UK.

### DNA substrates

M13 single-stranded DNA was from New England Biolabs. All DNA strands used to generate substrates are detailed in the Supplementary data (Table S1). Oligonucleotides (Sigma-Aldrich) were 5’ Cy5 end labelled for DNA annealing and primer extension reactions, and additionally with Cy3 for FRET-based DNA helicase assays. DNA substrates were annealed by heating a 1.2:1 ratio of unlabelled to Cy5-labelled oligonucleotides to 95°C for 10 min and annealing to room temperature overnight. Annealed DNA was separated from unannealed oligonucleotide by electrophoresis through 10% (w/v) acrylamide (37.5:1 acrylamide/bis*-*acrylamide was used throughout except if stated otherwise) gels comprising Tris-borate-EDTA (TBE), followed by excision of the desired substrate as a gel slice gel and soaking overnight into 50 mM Tris–HCl pH 7.5 containing 150 mM NaCl to recover DNA from gel pieces.

### Primer extension assays

Primer extension reactions (25 μl) were based on method in (33,34), utilizing purified human Pol δ (40 nM) in a holoenzyme complex with PCNA (40 nM), RFC (10 nM) and RPA (320 nM). RPA, PCNA and RFC were pre-mixed for 10 min on ice in the reaction buffer containing DNA substrate (82.5 ng M13-primer DNA/reaction) and dNTPs (200 μM), before adding Pol δ to start the reactions. Reactions were for 30 min at 37°C before adding 2 μl of STOP solution (50 mM Tris–HCl pH 8.0, 100 mM EDTA, 0.1% w/v SDS and 5 mg/ml of proteinase K). Cy5-labelled products after electrophoresis through a 0.8% agarose TBE gel were visualised using a Typhoon scanner, and unlabelled plasmid DNA was visualised by ethidium bromide staining and placing the gel on a UV transilluminator.

Primer extension reactions by isolated Pol δ complex (POLD1-D4) were in 20 μl containing substrate DNA (21 nt primer oligo annealed to a 70 nt oligo template, each at 15 nM), 10 mM MgCl_2_, 40 mM Tris–HCl pH7.5, 1 mM DTT, 0.2 mg/ml BSA, 50 mM NaCl and 200 μM of each dNTP. Primer extension assays using human DNA polymerase η and polymerase k were in buffer 40 mM Tris–HCl pH 8.0, 10 mM DTT, 0.25 mg/ml BSA, 60 mM KCl, 2.5% glycerol, 5 mM MgCl_2_, 200 μM dNTPs. Primer extension by *E. coli* polymerase III core (DnaE) and polymerase I were in buffer 10 mM magnesium acetate, 40 mM HEPES–NaOH pH 8.0, 0.1 mg/ml BSA, 10 mM DTT and 200 μM dNTPs. Unless stated otherwise, all polymerase primer extension assays were for 30 min at 37°C after adding DNA. Reactions were halted by adding 5 μl of stock STOP solution. Stopped reactions were mixed with loading dye (20% glycerol, 78% formamide and Orange G) for electrophoresis through 10% acrylamide (19:1 acrylamide: bis-acrylamide) TBE denaturing (8 M urea) gels, at 5 W for 3 h. Primer extension products were visualised *via* the Cy5-DNA end label using a Typhoon scanner, and files were quantified using ImageJ and Prism software. For primer extension reactions containing HelQ, proteins were pre-mixed in their storage buffers, in isolation from reaction buffer and DNA, and reactions commenced by adding buffer containing DNA and dNTPs.

### DNA annealing assays

These contained DNA strands detailed in Supplementary S1. For gel-based assays, reaction mixtures contained 20 mM Tris–HCl pH 7.5, 100 mg/ml BSA, 7% glycerol, 25 mM DTT, DNA strand ELB41 (15 nM). HelQ was added for incubation for 2 min at room temperature, and then annealing began by addition of 15 nM of DNA strand ELB40, for 5 min at 37°C when reactions were halted by adding 5 μl of STOP solution. Annealing products were assessed in 10% acrylamide (w/v) TBE gels electrophoresed for 1 h at 150 V.

FRET reactions contained 20 mM Tris–HCl pH 7.5, 100 mg/ml BSA, 7% glycerol, 5 mM DTT and 5’Cy5 labelled ELB41 (50 nM). HelQ was added for incubation for 2 min at room temperature, and then annealing began by addition of Cy3 labelled ELB40 (50 nM). Fluorescence was measured on a 37°C pre-incubated FLUOstar Omega (BMG Labtech) at excitation of 540 nm and emissions at 590 nm and 670 nM. Readings were taken every minute for 20 min. FRET values plotted are as a ratio of FRET values measured from a control reaction of fully annealed ELB40 and ELB41, and after subtracting background Cy3 fluorescence and FRET pair excitation in zero protein controls.

### DNA helicase assays and EMSAs

Gel-based helicase assays monitored liberation of Cy5 labelled single-stranded DNA from the forked DNA substrate—DNA strands used to generate the fork are listed in Supplementary Table S1. Reaction mixtures contained 20 mM Tris–HCl pH 7.5, 100 mg/ml BSA, 7% glycerol, 25 mM DTT, 5 mM ATP, 5 mM magnesium chloride and 25 nM Cy5 end-labelled DNA fork. Reactions were incubated at 37°C for 10 min prior to quenching by addition of STOP buffer. Reactions were resolved on 10% (w/v) acrylamide TBE gels and analysed using an Amersham Typhoon. Percentage unwinding was calculated in ImageJ by determining the percentage of Cy5 labelled ssDNA compared with Cy5 labelled forked DNA.

For FRET, reaction mixtures contained 20 mM Tris–HCl pH 7.5, 100mg/ml BSA, 7% glycerol, 5 mM DTT, 5 mM ATP, 5 mM magnesium chloride and 50 nM FRET pair labelled DNA fork substrate. On addition of protein, fluorescence was measured in a 37°C pre-incubated FLUOstar Omega (BMG Labtech) at excitation of 540 nm and emissions at 590 and 670 nm. Readings were taken every minute for 20 min. FRET values plotted are as a ratio of FRET values measured from a control reaction of DNA strands that are not unwound (zero protein) and after subtracting background Cy3 fluorescence.

Electrophoretic mobility shift assay (EMSAs) reaction mixtures contained 20 mM Tris–HCl pH 7.5, 100 mg/ml BSA, 7% glycerol, 25 mM DTT, 50 mM EDTA and 25 nM fluorescently labelled DNA and varying concentrations of HelQ and variant mutant proteins. Assembled complexes were incubated at 37°C for 10 min prior to resolution on 5% (w/v) acrylamide TBE gels in Orange G loading dye. Gels were imaged and analysed using an Amersham Typhoon phosphor-imager to detect migration of Cy5 end-labelled DNA.

### Bimolecular fluorescence complementation (BiFC)

DNA encoding the N-terminal fragment of the mVenus fluorescent protein (1–154aa, NmVenus) and C-terminal fragment of mVenus (155–238aa, CmVenus) were amplified from p63RhoGEF619-mVenus-N1 (Addgene) and cloned into pBADHisA via NhelI site (pBad-NmVenus) and pRSF-1b via NcoI (pRSF-CmVenus). We split the mVenus encoding gene at those sites because it was effective for a previous study in *E. coli* (35). The gene encoding full-length mVenus (positive control for BiFC assays) was amplified and cloned into pBADHisA via NheI and EcoRI sites. The genes encoding POLD3, POLD1, POLD2 and POLD4 were each PCR amplified from GeneArt *E. coli* optimised protein expression constructs for insertion *via via* XhoI and HinDIII sites in to pBad-NmVenus. The N-HelQ encoding gene was amplified from pTJ09 and inserted into pRSF-CmVenus *via* KpnI and XhoI sites. Each DNA construct was confirmed by DNA sequencing and expression of each protein was verified by SDS-PAGE (Figure S4i–iii) of cell samples taken during the BiFC assay.The BiFC assay was in *E. coli* BL21AI expressing an NmVenus-POLD subunit (as shown in results Figure 5) alongside CmVenus-N-HelQ. Overnight cultures (100 ul) of each were inoculated in 5 ml fresh LB and incubated at 37°C with shaking to OD_600_ of 0.5, when protein expression was induced by adding L-arabinose (0.05% w/v) and IPTG (1 mM). At each time point, 100 uL of cells were pelleted, resuspended in 150 ul of ice-cold PBS and transferred to a flat-bottom 96 well plate for imaging at 515–527 nm using the Typhoon™ laser-scanner platform and the built-in Cy2 filter system. Images were saved as TIFFs and presented using ImageJ.

## RESULTS

### HelQ halts DNA synthesis by DNA polymerase δ

**Figure 1.**
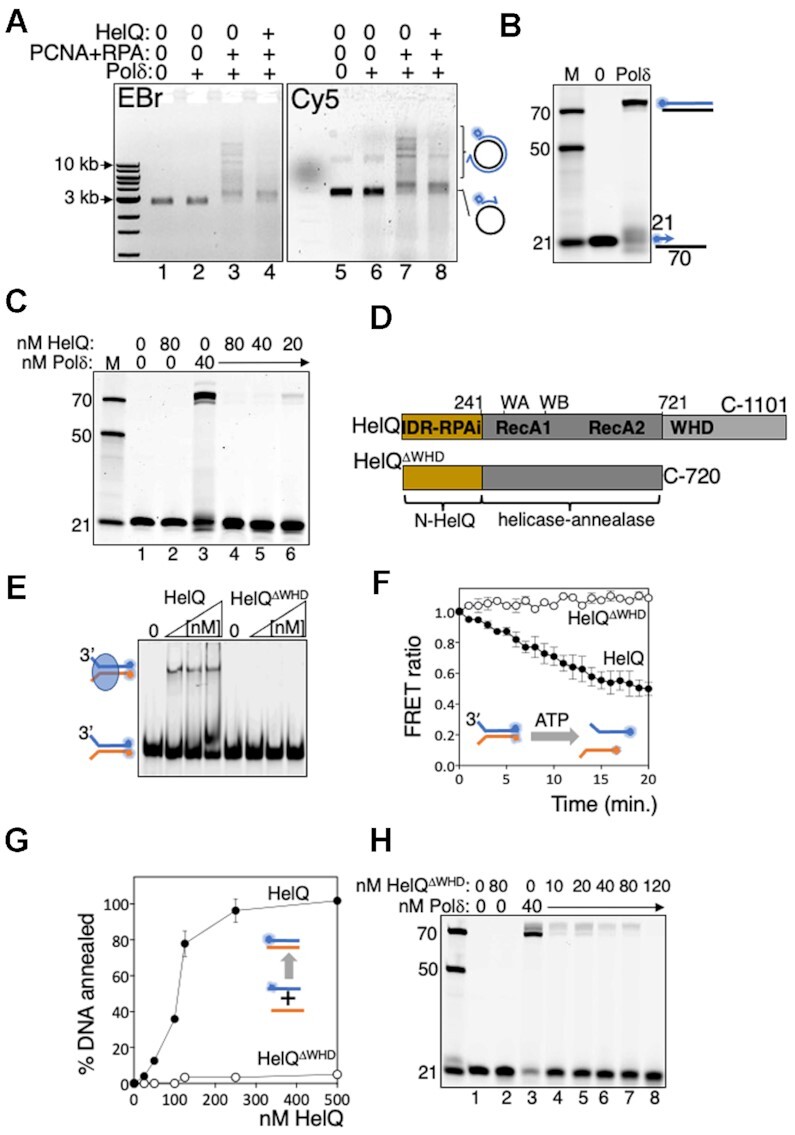
(**A**) Primer extension by Pol δ-RPA-PCNA holoenzyme is inhibited by addition of HelQ. Summarised in products from the M13 template visible in an ethidium bromide-stained agarose gel (EBr, lanes 1–4), and as extended Cy5-labelled primer (blue circle) from the same gel (Cy5, lanes 5–8). Proteins present in reactions are indicated (+); Pol δ (40 nM), PCNA (40 nM), RPA (320 nM), RFC (10 nM) and HelQ (100 nM) were added to M13 DNA (82.5 ng) pre-annealed with a 44 nt Cy5-M13 primer. (**B**) Pol δ complex alone (40 nM) extends a Cy5 (blue circle) labelled 21nt primer (15 nM) against a 70nt template, visualised in a denaturing (8 M urea) gel. Cy5 labelled DNA markers are indicated on the panel left (21, 50 and 70nt), and in subsequent gel panels. To the panel right denotes fully extended product of Pol δ visible in the urea gel. (**C**) DNA synthesis by Pol δ (40 nM) was inhibited by titration of HelQ (nM as indicated), lanes 4–6, compared with uninhibited DNA synthesis (lane 3) and including control reactions (lanes 1 and 2) with proteins included as indicated. (**D**) HelQ^ΔWHD^ has intact the catalytic helicase-annealase domains (RecA1 and A2), and the RPA inhibiting (RPAi (23)) intrinsically disordered region (IDR, see also Figure 2), but lacks the predicted winged helix domain (WHD) (23,36). (**E**) EMSA showing HelQ^ΔWHD^ (100, 200, 400 nM) is unable to detectably bind to a forked DNA substrate (15 nM) that is stably bound by HelQ (100, 200, 400 nM). (**F**) FRET measurements comparing unwinding of forked DNA (15 nM) by HelQ^ΔWHD^ and HelQ (each 150 nM). Reduced FRET ratios correspond to separation of Cy3-Cy5 pairs during DNA unwinding. (**G**) HelQ^ΔWHD^ is severely deficient in DNA strand annealing compared with HelQ measured from end-point assays (*n* = 3, bars are standard error) for pairing of complementary DNA strands. (**H**) HelQ^ΔWHD^ (nM as indicated) inhibits DNA synthesis by Pol δ (40 nM, lane 3), summarised in an acrylamide–urea gel.

We investigated if HelQ modulates DNA synthesis reactions *in vitro* as a possible explanation for genetic instability in *HELQ^−^^/^^−^* cells (28). Purified HelQ protein (Figure S1A) was introduced into primer extension reactions catalysed by Pol δ. Purified human Pol δ comprising four subunits (Figure S1A, POLD1/p125, POLD2/p50, POLD3/p68 and POLD4/p12 (31)) forms a complex with PCNA and RPA for processive DNA synthesis—in our assays this was apparent from Pol δ (40 nM) requiring pre-incubation with RPA and PCNA to synthesise DNA against M13 as a template, from a 44nt Cy5 end labelled primer, summarised in Figure 1A (compare lane 2 with 3, and lane 6 with 7 and 5–7). DNA replication products of Pol δ-RPA-PCNA holoenzyme visible in size from 3 to >10 kb on ethidium bromide staining and Cy5 imaging (Figure 1A, lanes 3 and 7), were robustly inhibited by adding HelQ (100 nM) after pre-incubation of Pol δ with RPA-PCNA (Figure 1A lanes 4 and 8). One potential explanation for this was the ability of HelQ to remove RPA from ssDNA shown in previous work (23,29), which could potentially inhibit DNA synthesis in these reactions by depriving Pol δ of RPA. We therefore switched to primer extension reactions in which Pol δ synthesises DNA against a short oligonucleotide template, without need for RPA and PCNA (Figure 1B and Supplementary Figure S1B). Again we observed inhibition of Pol δ (40 nM) to zero detectable product formation on titrating HelQ (20–80 nM) into the reactions immediately after addition of Pol δ to DNA, summarised in Figure 1C (compare lane 3 to lanes 4–6).

HelQ binding to ssDNA activates its DNA translocation/helicase activity (22,23). This raised a further possible explanation for its inhibitory effect on Pol δ, if it physically prevented primer extension as a barrier or by removing Pol δ. To test this we isolated a novel HelQ protein (HelQ^ΔWHD^, Figure 1D and Supplementary Figure S1A) that lacks a predicted DNA-binding winged helix domain (36). HelQ^ΔWHD^ was unable to bind to DNA in EMSAs (summarised in Figure 1E) and was correspondingly inactive at DNA unwinding and DNA annealing, measured in real time by FRET efficiencies and confirmed from products observed in gels at assay end points (Figure 1F and G, and Supplementary Figure S1C and D). HelQ^ΔWHD^ halted primer extension by Pol δ (Figure 1H), therefore we concluded that HelQ halts DNA synthesis by Pol δ by a mechanism independent from interaction with RPA or DNA.

### An intrinsically disordered region of HelQ selectively inhibits pol δ

**Figure 2.**
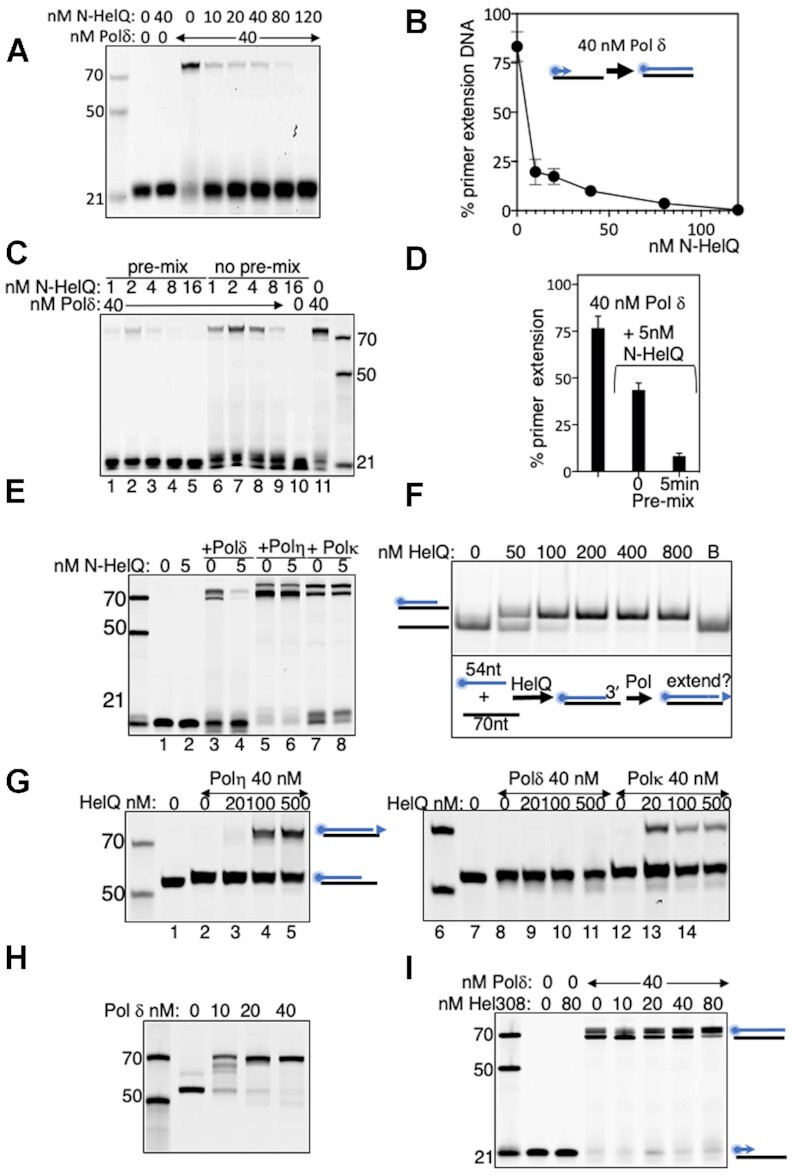
(**A**) DNA synthesis by Pol δ (40 nM, lane 3) inhibited by titration of N-HelQ (nM as indicated), and in (**B**) quantified where *n* = 3 showing standard error from mean. (**C**) DNA synthesis by Pol δ (lane 11, 40 nM) inhibited by N-HelQ (nM as indicated) most effectively by pre-incubation with Pol δ for 5 min prior to addition of DNA (lanes 1–5), compared with adding the same concentration of proteins separately to DNA simultaneously (lanes 6–10). In (**D**), this is quantified for Pol δ (40 nM) and N-HelQ (5nM) with either pre-mixing of proteins prior to adding DNA, or not. *N* = 3, showing standard error from mean. (**E**) DNA synthesis by Pol δ (60 nM) inhibited by N-HelQ (5 nM) (lanes 3 and 4) whereas other polymerases indicated were unaffected (lanes 5–8, and Supplementary figures). (**F**) Native acrylamide (TBE) gel showing HelQ (nM as indicated) annealing a 70nt DNA strand with a complementary cy5-end labelled 54nt DNA strand to generate a recessed 3’ DNA end that can be potentially extended by a polymerase, illustrated in the scheme below the gel. Lane B is boiled duplex to release the cy5 end labelled ssDNA. (**G**) Urea gels of HelQ-dependent primer extension products generated by human DNA polymerases η (lanes 1–5) and κ (lanes 11–14) but not Pol δ (lanes 3–5), despite (**H**) Pol δ extending the same DNA when annealed using heat instead of HelQ. (**I**) Summary that the archaeal homologue of HelQ, which lacks the N-HelQ region, is unable to inhibit Pol δ in primer extension reactions (nM as indicated).

To learn more about the inhibitory mechanism we turned to the region of HelQ comprising amino acids 1–241, termed N-HelQ (see Figure 1D), because it is unable to bind to DNA and was previously shown to utilize a small protein fold to modulate RPA function (23). N-HelQ also inhibited Pol δ (40 nM), reducing primer extension to zero at 120 nM (Figure 2A and B). Inhibition by N-HelQ was enhanced at least 5-fold by pre-mixing it with Pol δ for 5 min before adding DNA to reactions (1–16 nM N-HelQ: 40 nM Pol δ) (Figure 2C, D and Supplementary Figure S2A). N-HelQ was selective at inhibiting DNA synthesis by Pol δ—human polymerase η (Pol η) and polymerase κ (Pol κ), and bacterial polymerases I and III, were all unaffected by addition of N-HelQ to primer extension reactions (Figure 2E and Supplementary Figure S2B). These data are consistent with inhibition requiring a specific protein–protein interaction.

We therefore tested whether catalytically active full length HelQ was also selective for targeting Pol δ. For this we utilized DNA annealing by HelQ (29), to couple demonstrable HelQ function with it generating a substrate available for primer extension by a DNA polymerase. HelQ annealed DNA strands of unequal length (54 and 70 nucleotides) in our assays, creating product with a recessed duplex 3’ end with potential for primer extension (Figure 2F). These annealing reactions, and controls lacking HelQ but containing DNA, were mixed without de-proteinising with primer extension buffer containing Pol η, Pol κ or Pol δ (40 nM). Pol δ failed to generate primer extension products from HelQ-annealing reactions (Figure 2G, lanes 6–10), despite extending the DNA primer after it was annealed by heating-cooling instead of by HelQ (Figure 2H), but Pol η and Pol κ were effective at DNA synthesis triggered by HelQ annealing (Figure 2G lanes 1–5 and 11–14). Finally, we confirmed that the N-HelQ region is required for full inhibition of Pol δ when observing that the Hel308 helicase-annealase—a close sequence homologue of the HelQ catalytic domains but which lacks the N-HelQ region (37)—did not inhibit DNA Pol δ (Figure 2I), and that a mutant HelQ protein (C-HelQ (23)) that lacks the N-HelQ region is less effective than N-HelQ or full HelQ at inhibiting Pol δ despite being fully proficient at binding to DNA (Figure S2B).

### Distinct regions of HelQ inhibit DNA synthesis by pol δ and DNA binding by RPA

**Figure 3.**
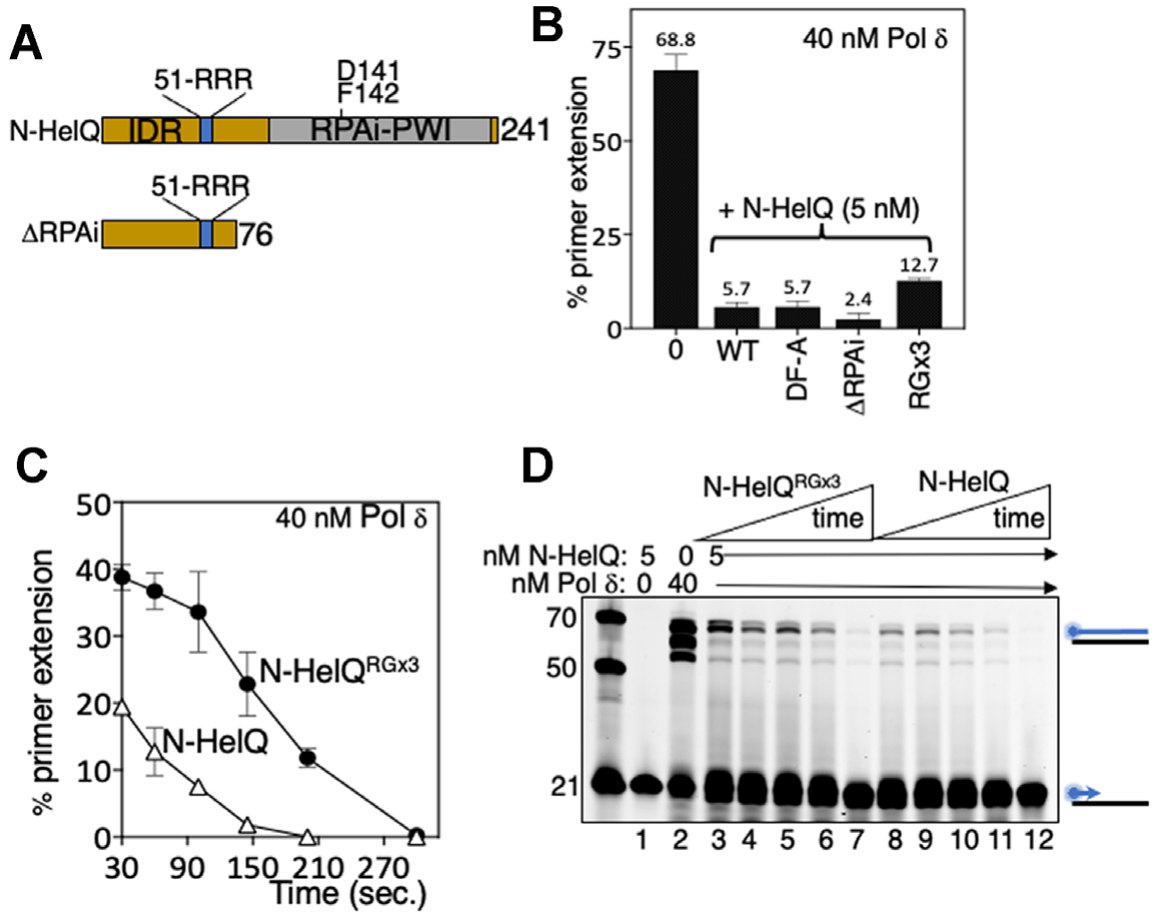
(**A**) Schematic summarising landscape of N-HelQ and mutant forms we generated to test inhibition of Polδ. Residues R51–R52–R53 in the intrinsically disordered region of N-HelQ were mutated alongside D141 and F142 that are required to modulate RPA, located in a predicted PWI-like fold (23). The 76-amino acid N-HelQ^ΔRPAi^ fragment that is intrinsically disordered was fully proficient at inhibiting Pol δ. (**B**) End point measurements of inhibition of DNA synthesis by Pol δ (40 nM, maximally in these assays 68.8% of DNA as product) caused by unmutated N-HelQ (5.7%) compared with mutants (5 nM) as indicated (‘DF-A’ is N-HelQ D142AF143A; ΔRPAi, is the N-HelQ 76 amino acid fragment; RGx3 is N-HelQ R51G/R52G/R53G). N-HelQ^RGx3^ protein showed comparatively reduced inhibition of Pol δ in these assays (12.7%) (*n* = 3, showing standard error from the mean). (**C**) Measurements of inhibition of DNA synthesis by Pol δ (40 nM) by N-HelQ or N-HelQ^RGx3^, pre-mixing the proteins for 30 s to 5 min, before adding DNA substrate and dNTPs. The graph shows data points with standard error of mean (*n* = 3). A representative gel is shown in (**D**).

Having established that catalytically active HelQ and its non-catalytic N-HelQ fragment both inhibit Pol δ we generated mutant N-HelQ proteins to map residues required for inhibition. We focussed on two distinct regions of N-HelQ—the PWI-like protein fold (amino acids 128–237) that disrupts RPA–ssDNA complexes through conserved Asp-141 and Phe-142 residues (Figure 3A, RPAi-PWI) (23), and a region (amino acids 1–76) that gives no predicted structural elements in modelling algorithms. Pol δ mixed with N-HelQ or N-HelQ mutants for 5 min prior to adding DNA, showed that N-HelQ mutated in Asp-141 and Phe-142 (N-HelQ^DF-A^) inhibited Pol δ similarly to unmutated N-HelQ (Figure 3B). In agreement with this, a 76 amino acid N-HelQ fragment lacking any of the RPA-interacting PWI fold (N-HelQ^ΔRPAi^, Figure 3A), was fully proficient at inhibiting Pol δ (Figure 3B). These 76 amino acids of N-HelQ are poorly conserved in HelQ proteins across species except for a tract of basic amino acids that show some conservation (in human HelQ, arginines-51, -52 and -53, and lysine-54, Figure 3A and Supplementary Figure S2D). Changing all three arginines to glycine (N-HelQ^RGx3^) resulted in a modest but reproducible decrease in inhibition of Pol δ in end point assays (Figure 3B). In the same reactions as a function of time—pre-mixing N-HelQ^RGx3^ with Pol δ from 30 to 300 s prior to adding DNA—gave substantially less inhibition of Pol δ compared with unmutated N-HelQ (Figure 3C and D). The data together indicates that inhibition of DNA synthesis by Pol δ maps to the intrinsically disordered extreme 76 amino acids of the HelQ N-terminus, distinct from that removing RPA from ssDNA.

### HelQ functionally and physically interacts with POLD3, and does not halt DNA synthesis by POLD1

**Figure 4.**
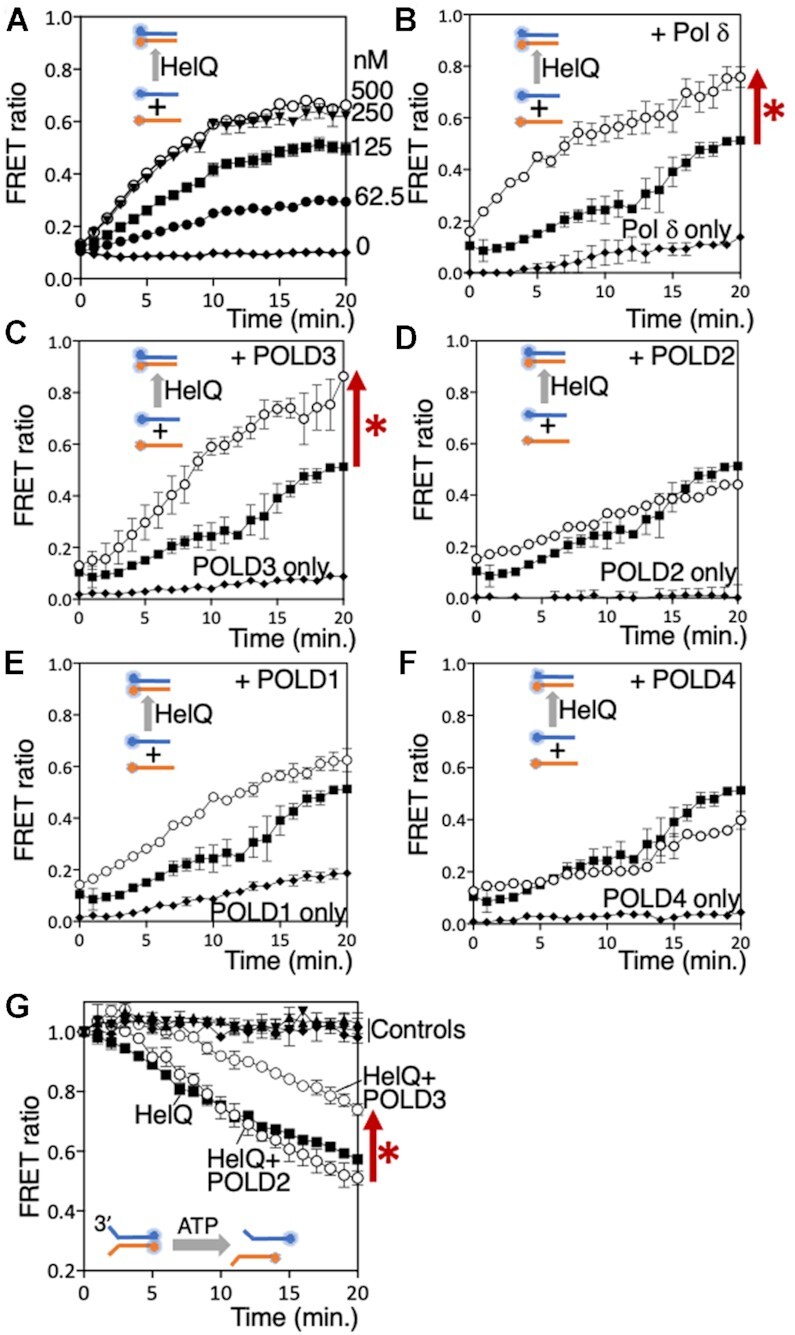
(**A**) Real-time measurement of FRET from HelQ (nM as indicated) annealing two Cy5/Cy3 end-labelled complementary 70nt DNA strands by HelQ at the nM concentrations indicted. (**B–F**) Each graph shows measurement of FRET (*n* = 2, standard deviation bars) from DNA annealing of the Cy3/Cy5 complementary DNA strands. In each panel—black squares for HelQ alone (125 nM); white circles HelQ with addition of either Pol δ, POLD1, POLD2, POLD3 or POLD4 (150 nM, as indicated); black diamonds for reactions lacking HelQ but containing only either Pol δ or POLD1, -2, -3 or -4. In each panel, the three plots are after adjusting for FRET measurement from zero protein control reactions, and the FRET measurement is in ratio to 100% (1.0) annealing from a fully base-paired control reaction. The red arrow and asterisk next to panels B and C highlight HelQ annealing that was stimulated by Pol δ or POLD3. (**G**) FRET measurement of HelQ-catalysed unwinding of a DNA substrate (15 nM) by HelQ (50 nM, black squares) and with addition of POLD2 (50 nM, labelled as white circles) or POLD3 (50 nM, also labelled). Controls, which are labelled, all showed no decrease in FRET indicating no DNA unwinding and were no protein reactions (black diamonds), POLD3 only (black triangles) and POLD2 only (inverted black triangles). POLD4 is shown in Supplementary Figure S3B for clarity. Next to the graph the red arrow and asterisk highlights the effect of adding POLD3 to helicase unwinding by HelQ.

We next focussed on Pol δ and its individual subunits (Figure S1A) to learn more about interactions with HelQ, first testing for modulation of HelQ. HelQ (62.5–500 nM) catalysed annealing of complementary 70-nt DNA strands (15 nM each) measured by FRET in real time (0–20 min, Figure 4A), and observed as end-point products in gels (Supplementary Figure S3A). HelQ (125 nM) stimulated the measured FRET ratio—proportion of DNA annealed by HelQ compared with fully annealed duplex (1.0) DNA (see Materials and Methods)—from a base-line of 0.11 in controls lacking HelQ to ratio of 0.5 (Figure 4A), indicating HelQ-dependent annealing of 41% of available DNA strands. These conditions were carried forward into parallel reactions adding Pol δ or individual POLD subunits. Reactions adding Pol δ complex (150 nM) with the second DNA strand stimulated annealing, increasing FRET from 0.51 to 0.76 (Figure 4B). Control reactions containing only Pol δ complex showed significant annealing (maximally a ratio of 0.14, Figure 4B), although this accounted for only half of the increased annealing by HelQ-Pol δ. DNA annealing by HelQ was much enhanced when POLD3 alone was added—FRET efficiency in these reactions increased from 0.50 to 0.87 against base-line FRET of 0.07 from POLD3 alone (Figure 4C). This functional interaction between POLD3 and HelQ contrasted with no significant effect, after adjustment for reactions containing no protein or only proteins Pol δ, POLD1, POLD2 or POLD4 alone without HelQ (Figures 4D–F). HelQ helicase unwinding was significantly inhibited by POLD3, in-line with POLD3 instead promoting the opposite reaction, DNA annealing. In helicase assays HelQ (50 nM) decreased the mean FRET efficiency from 0.98 for fully paired DNA strands to 0.57, as FRET pairs are separated by DNA unwinding (Figure 4G). Addition of POLD3 increased FRET to 0.74, but POLD2 and POLD4 had no inhibitory effect, reactions giving mean FRET efficiencies of 0.51 and 0.54 respectively (Figure 4G and Supplementary Figure S3B). Inclusion of POLD1 or Pol δ in these assays was not possible ATP-Mg^2+^ (5 mM each) strongly stimulated their 3’ to 5’ exonuclease activity, which degrades the DNA substrate from the 3’ end that is required for HelQ to load and translocate (22,23). However, we conclude that POLD3 is effective at modulating DNA processing by HelQ.

**Figure 5.**
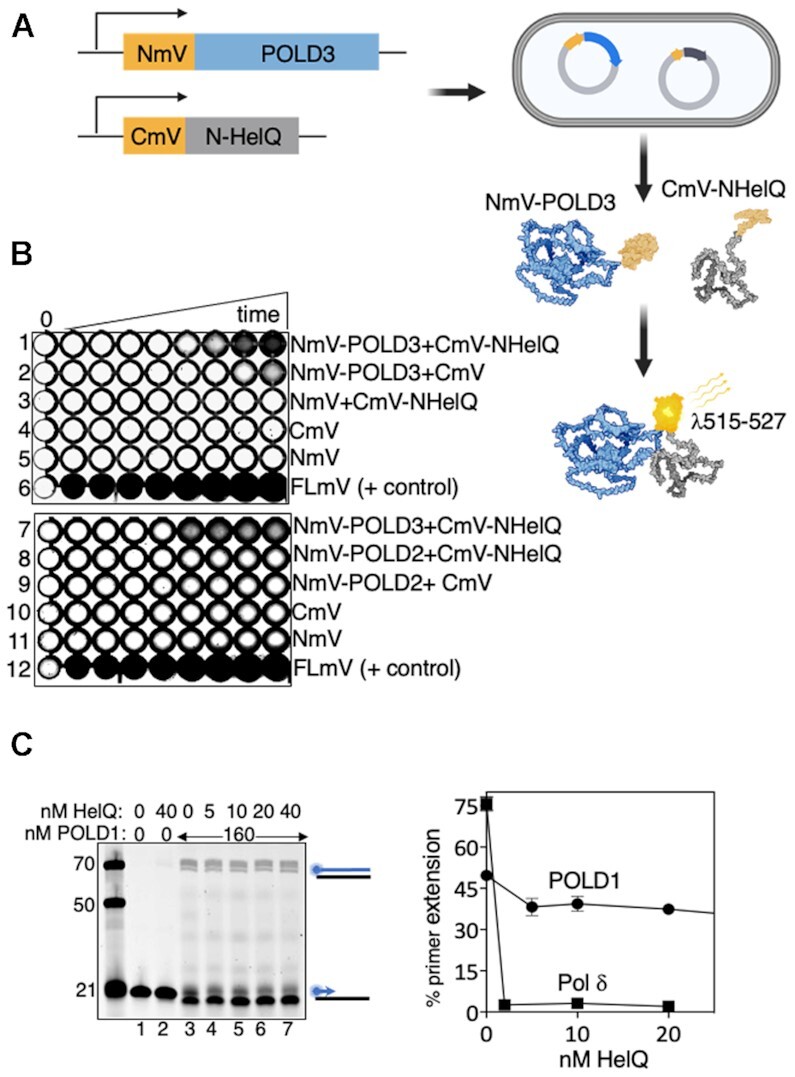
(**A**) Schematic of the BiFC assay to detect physical interaction between proteins. N-terminal and C-terminal fragments of mVenus protein (NmV and CmV) were fused to POLD3 or N-HelQ in expression vectors for *E. coli*. NmV and CmV are reconciled if POLD3 and N-HelQ physically interact, detectable by constitutive fluorescence of mVenus. (**B**) mVenus (mV) fluorescence was detectable in 96-well plates from *E. coli* cell samples co-expressing the NmV-POLD3 fusion alongside CmV-N-HelQ as indicated (row 1). N-HelQ was utilised in preference to full length human HelQ, which does not over-express in *E. coli*. Other co-expression control rows are as indicated; NmV-POLD3 with CmV, CmV-N-HelQ with NmV, CmV and NmV alone, and a positive control for continuous fluorescence from full length mV (FLmV). Time points (5, 15, 30, 60, 90, 120, 150 and 180 min) were from induction of protein expression. (**C**) Summary showing that HelQ (nM as indicated) is unable to inhibit DNA synthesis by the POLD1 (160 nM) catalytic subunit of Pol δ, and in (**D**) measured (*n* = 3, bars of standard error) in comparison with inhibition of Pol δ.

Stable physical interaction between POLD3 and HelQ or N-HelQ could not be detected in SEC, affinity protein pull-downs, SPR or EMSAs, therefore we turned to the more sensitive in-cell method of bimolecular fluorescence complementation (BiFC) (38,39) to test for physical interaction between POLD3 and HelQ during their co-expression in *E. coli*. For BiFC, summarised in Figure 5A, the constitutively fluorescent mVenus protein is bisected and each part (N-mVenus and C-mVenus) fused to a protein of interest (35). Proximity of N-mVenus and CmVenus protein fragments brought about by physical interaction of their fused proteins causes mVenus to fluoresce, detectable at 515–527 nm in cells placed in 96-well plates. Inducible co-expression in *E. coli* cells of N-mVenus fused to POLD3 (NmV-POLD3) alongside C-mVenus fused to N-HelQ (CmV-N-HelQ) triggered fluorescence from 90 min, indicating POLD3-N-HelQ interaction (Figure 5B row 1). Fluorescence was not observed from co-expression of standard N-mVenus/CmVenus control protein pairings (Figure 5B, rows 2–5), or in independent assays when NmV-POLD3 was substituted for NmV-POLD2 (Figure 5B, compare rows 7 and 8), or for NmV-POLD1 or NmV-POLD4 (Supplementary Figure S4A), despite fused proteins all expressing detectably in cells (Figure S4B). This data from BiFC is consistent with functional interaction between HelQ and POLD3 observed in DNA annealing and helicase assays (Figure 4).

POLD3 interacts with POLD1 in human Pol δ to regulate DNA synthesis during homologous recombination. Isolated POLD1 can catalyse DNA synthesis against short oligonucleotide templates (Figure 5C, lane 3), but intriguingly neither HelQ or N-HelQ inhibited POLD1 (Figure 5C, lanes 4–7, graph and Supplementary Figure S5)—primer extension products of POLD1 (160 nM) were maintained at 49% of total DNA, compared with Pol δ complex that was inhibited from 75% of DNA product to zero, as expected. This is consistent with physical interaction between the intrinsically disordered region of HelQ and the POLD3 subunit of Pol δ acting as the control point for inhibiting of DNA synthesis and stimulating DNA single strand annealing.

## DISCUSSION

**Figure 6.**
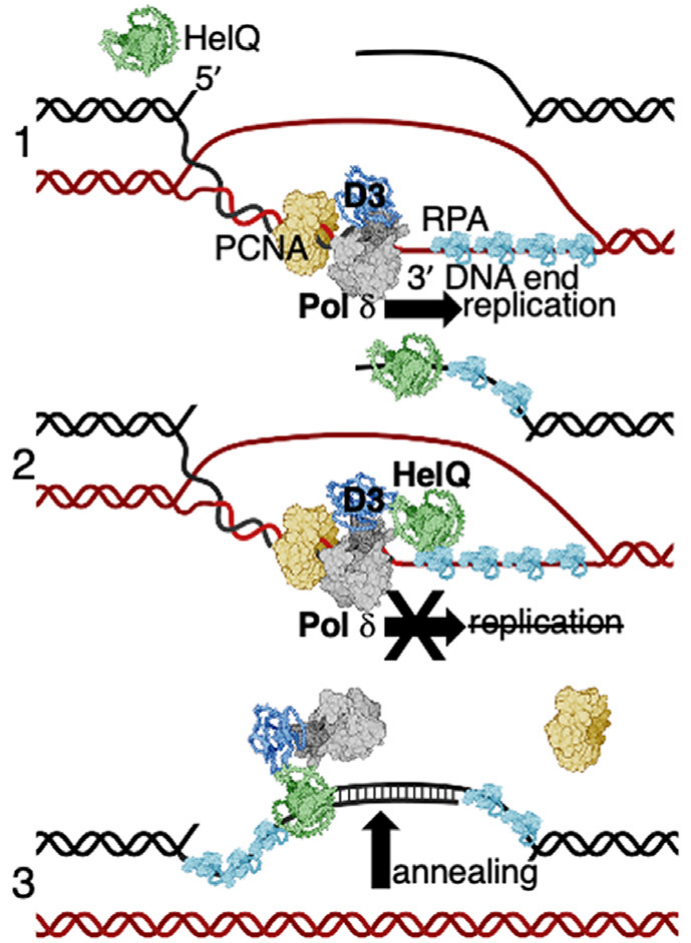
A model illustrating HelQ restraining DNA synthesis by Pol δ during homology-directed DNA repair, promoting POLD3-stimulated DNA strand annealing. For simplicity HelQ (green) is shown as a monomer—no atomic resolution structure of HelQ is yet available. 1) The 3‘single strand end of broken DNA (black) is paired with unbroken DNA (red) that primes DNA replication in the direction of the arrow, catalysed by polymerase δ (Pol δ grey/blue) in complex with PCNA (yellow) and RPA (sky blue). 2) Interaction of HelQ with POLD3 of the Pol δ complex halts DNA replication—HelQ interacts with RPA and displaces RPA from DNA (23,29), which may orientate HelQ to sites of DNA synthesis by Pol δ. 3) Interaction of HelQ with POLD3 stimulates HelQ to anneal nascent single-stranded DNA synthesised by Pol δ with its parent DNA duplex (black). The mechanism of annealing is not known, but is proficient with or without ATP hydrolysis by HelQ. These processes are proposed to limit the extent of mutagenic DNA synthesis during DNA repair in metazoans.

Our identification that HelQ-Pol δ interaction robustly inhibits DNA synthesis and stimulates DNA single-strand annealing provides new insight into how HelQ maintains genome stability, and the genetic defects arising in *HELQ^−^^/^^−^* cells (25,26,28,29). Tandem DNA duplications and long tract recombination from DNA break repair are characteristic of chronic DNA damage triggering unrestrained DNA synthesis by Pol δ during break-induced replication, microhomology-mediated break induced replication, and synthesis-dependent strand annealing (6,11,40–43). Previous work revealed that HelQ removes RPA from ssDNA (23,29), and that ssDNA triggers ATP-dependent DNA translocation by HelQ (22,23), which is further stimulated by Rad51 (29). Therefore, alongside the data presented here, a model emerges for HelQ as a key controller of the extent of homologous recombination; by assembling onto ssDNA *via* RPA for ATP-dependent DNA translocation until it encounters Pol δ, halting DNA synthesis and triggering single-strand DNA annealing, summarised in Figure 6.

A polypeptide fragment at the extreme N-terminal end of HelQ (N-HelQ amino acids 1–76) was fully proficient at inhibiting Pol δ, an effect diminished by mutating a tract of arginine residues (Arg51–53). In contrast, substitution of residues (Asp-141 and Phe-142) in N-HelQ that are critical for HelQ destabilising RPA-DNA complexes had no effect on inhibition of DNA synthesis by Pol δ. Therefore N-HelQ seems to provide a ‘hub’ for controlling at least RPA and Pol δ in distinct protein-protein interactions.

Our observation of functional and physical interaction between POLD3 and HelQ/N-HelQ, and the impotence of HelQ or N-HelQ against DNA synthesis by POLD1 alone, further highlight a plausible mechanism for how HelQ may control DNA repair synthesis. POLD3, alongside Pif1 helicase, promotes DNA synthesis during human homologous recombination by Break-Induced Replication (44), where it is thought to stabilise the Pol δ complex. POLD3 is also required for DNA synthesis by the REV3L subunit of human polymerase ζ complex that drives mutagenic DNA synthesis in several contexts, including Microhomology-Mediated Break Induced Replication (45). The recent insights from genetics alongside new biochemical data presented here advances a model for HelQ protein as a restraint to DNA synthesis arising from unproductive homologous recombination and other DNA homology-dependent repair processes.

## DATA AVAILABILITY

The data underlying this article are available in the article and in its online supplementary material.

## Supplementary Material

gkad032_Supplemental_Files
