# Supplementary material for: Interaction of human HelQ with DNA polymerase delta halts DNA synthesis and stimulates DNA single-strand annealing
Source: Nucleic Acids Res. 2023 Jan 31;51(4):1740–9. doi: 10.1093/nar/gkad032 (PMC9976902; doi:10.1093/nar/gkad032)
Supplement: gkad032_Supplemental_Files [file gkad032_supplemental_files.zip › R1 Supplementary Data File.docx]

**Supplementary Table S1**

DNA substrates used in this work for EMSAs, primer extension DNA annealing and DNA unwinding reactions.

| **DNA** | **5’ to 3’ –Cy5 or Cy3 end-labelled as indicated in results** | **Usage** |
| --- | --- | --- |
| Cy5-M13 | CAGGGTTTTCCCAGTCACGACGTTGTAAAACGACGGCCAGTGCC | Primer extension against M13 template |
| ELB40-v-oligo | GGAGCTCCCTAGGCAGGATCG | Primer extension against oligonucleotide 70mer |
| ELB40 | GGAGCTCCCTAGGCAGGATCGTTCGCGACGATGGCCTTC GAAGAGCTCCAGTTACGGATACGGATCCTGC | Annealing - Complement of ELB41 |
| ELB41 | GCAGGATCCGTATCCGTAACTGGAGCTCTTCGAAGGCCATCGTCGCGAACGATCCTGCCTAGGGAGCTCC | Primer extension and annealing |
| PM6B | TAAGAGCAAGATGTTCTATA**AAAGATGTCCTAGCAAG** | Primer extension D-loop; ‘invading’ strand. **Bold** nucleotides pair with RGL19. |
| PM4 | GGGTGAACCTGCAGGTGGGCGGCTGCTCATCGTAGGTTAGTTGGTAGAATTCGGCAGCGTC | Primer extension D-loop; displaced ‘loop’ strand |
| ELB40-B | GGAGCTCCCTAGGCAGGATCGTTCGCGACGATGGCCTT CGAAGAGCTCCAGTTA | Complement for 54/70 ELB41 – generates recessed 3’ end |
| Cy3-ELB40 | GGAGCTCCCTAGGCAGGATCGTTCGCGACGATGGCCTTCGAAGAGCTCCAGTTACGGATACGGATCCTGC | 3’ Cy3 for FRET assays, fully complimentary |
| Cy3-/Cy5 MW12 | GTCGGATCCTCTAGACAGCTCCATGATCACTGGCACTGGTAGAATTCGGC | DNA helicase fork substrate |
| Cy3-/Cy5  MW14 | CAACGTCATAGACGATTACATTGCTACATGGAGCTGTCTAGAGGATCCGA |  |

**Supplementary Figures**

**Figure S1A**

Coomassie stained SDS-PAGE gels of purified proteins used in this work alongside protein size markers. RPA protein used was the same as shown in [1].

**Figure S1B**

Urea gel summarising time course (0, 1, 2, 5, 10, 15, 20 and 30 minutes) DNA synthesis by purified Pol δ (40 nM) observed as extension of a 21 nucleotide (nt) cy5-labelled DNA annealed to a 70 nt template. Known length cy5 DNA markers are shown to the left of the panel, and in subsequent panels.

**Figure S1C**

Native TBE gel showing helicase unwinding products from a DNA fork substrate (15 nM) of HelQ compared with no unwinding from HelQ^ΔWHD^ at concentrations indicated.

**Figure S1D**

Native TBE gel showing HelQ or HelQ^ΔWHD^ DNA single strand annealing products from two complementary 70 nucleotide strands (each 15 nM). Protein concentrations for this assay were 200, 400, 600, 800 and 1000 nM to test the mutant HelQ to the maximum concentration possible.

**Figure S2A**

Urea gel showing primer extension by Pol δ (40 nM) in 30-minute reactions when in the absence of pre-mixing with N-HelQ (lane 2) or after pre-mixing with N-HelQ (10 nM) for periods of time as indicated; 30, 60, 90, 120, 180, 240, 300, 420 and 600 seconds (lanes 3-11) prior to adding DNA to the proteins.

**Figure S2B**

Urea gel summarising that N-HelQ (10 nM) has no effect on DNA synthesis by primer extension catalysed by *E. coli* DNA polymerases PolI and PolIII (each 80 nM), with DNA length markers as indicated.

**Figure S2C**

Urea gels summarising inhibition of Pol δ (40 nM) primer extension reactions, comparing HelQ, N-HelQ and C-HelQ (all 20 nM). Inhibition summarised for C-HelQ (lane 9) is reduced compared with N-HelQ and HelQ (lanes 3 and 6), and C-HelQ also modestly inhibits *E. coli* DNA polymerase III core enzyme, indicating inhibition of both polymerases non-specifically by C-HelQ, accounted for by ssDNA DNA binding.

**Figure S2D**

Alignment using Clustal Omega of amino acids from the N-HelQ region of HelQ (residues 1-271) from human (Hs), mouse (Mm), zebrafish (Dr) and the nematode *C. elegans* (Ce), as indicated. Highlighted in blue is the N-HelQ^ΔRPAi^ region with the conserved tract of basic residues described in the main text.

**Figure S3A**

Native TBE gel summarising DNA annealing by HelQ (concentrations as indicated) corresponding to FRET measurements.

**Figure S3B**

FRET measurements of HelQ DNA helicase activity that is the same data as in Figure 4G but with plot lines for POLD4 added (brown circles) showing no inhibition of HelQ helicase activity, similarly to POLD2.

**Figure S4A**

mVenus (mV) fluorescence in 96-well plates from *E. coli* cell samples co-expressing the NmV- Time points were after induction of cells for protein expression at 5, 15, 30, 60, 90, 120, 150 and 180 minutes.

**Figure S4B (i-iii)**

Protein over-expression corresponding to the BiFC assays, showing that control mVenus proteins and N-HelQ, POLD1, POLD2, POLD3, and POLD4 mVenus-fusion proteins were all over-expressed in cells during the assay after 2 hours of cell growth. The gels are 12.5% acrylamide SDS-PAGE stained with Coomassie. Abbreviations show are: Full-length mVenus protein (FLmV); N- or C-terminal mVenus (NmV, CmV).

**Figure S5**

Urea gel summarising that basal-level primer extension by POLD1 (lane 3, 160 nM as indicated) is unaffected by titration of N-HelQ as indicated, in agreement with data in Figure 3 using full HelQ.

**Supplementary Reference**

1. Jenkins, T., et al., *The HelQ human DNA repair helicase utilizes a PWI-like domain for DNA loading through interaction with RPA, triggering DNA unwinding by the HelQ helicase core.* NAR Cancer, 2021. **3**(1): p. zcaa043. doi: 10.1093/narcan/zcaa043
