# Supplementary material for: Interaction of human HelQ with DNA polymerase delta halts DNA synthesis and stimulates DNA single-strand annealing
Source: Nucleic Acids Res. 2023 Jan 31;51(4):1740–9. doi: 10.1093/nar/gkad032 (PMC9976902; doi:10.1093/nar/gkad032)

S1A

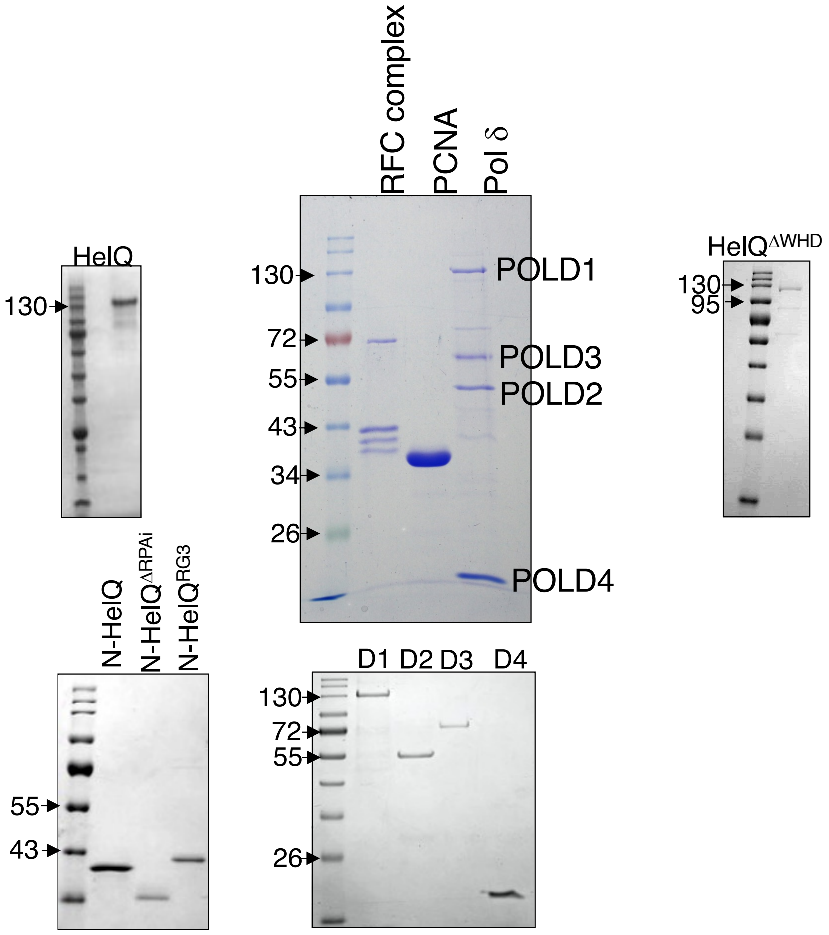
HelQ
130
RFC complex
PCNA
Pol δ
130
72
55
43
34
26
POLD1
POLD3
POLD2
POLD4
HelQ$^{\Delta WHD}$
130
95
N-HelQ
N-HelQ$^{\Delta RPAi}$
N-HelQ^{RG3}
55
43
D1
D2
D3
D4
130
72
55
26

S1B

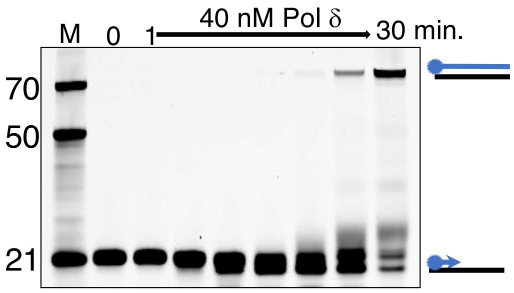
M
0
1
40 nM Pol δ
30 min.
70
50
21

S1C

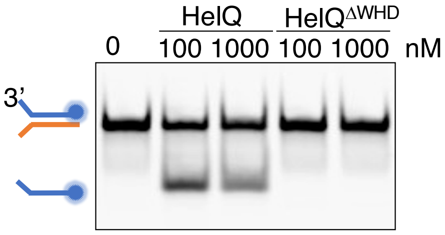
HelQ
HelQ$^{\Delta WHD}$
0
100 1000 100 1000 nM
3'

S1D

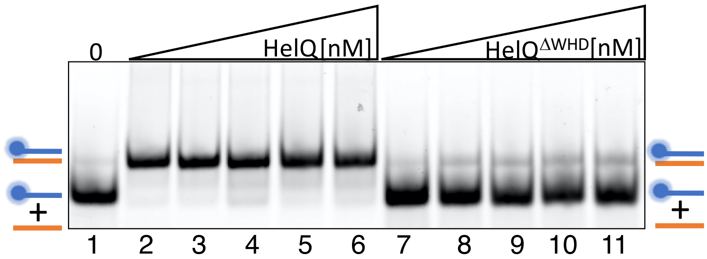
0
HelQ[nM]
HelQ$^{\Delta WHD}$[nM]
+
+
1 2 3 4 5 6 7 8 9 10 11

S2A

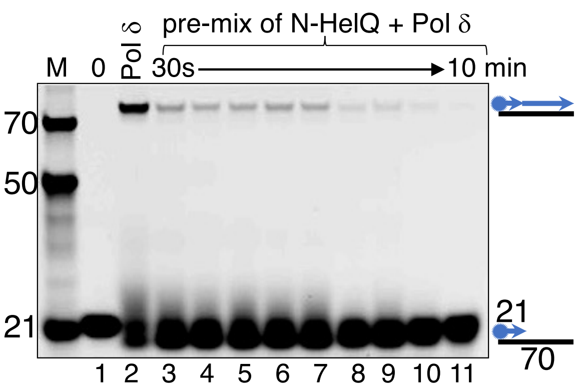

S2B

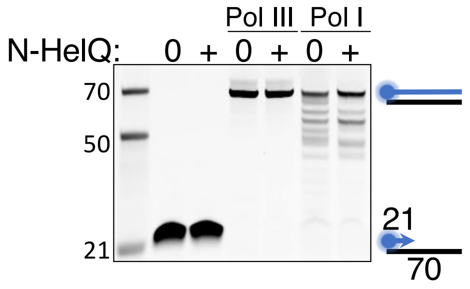

S2C

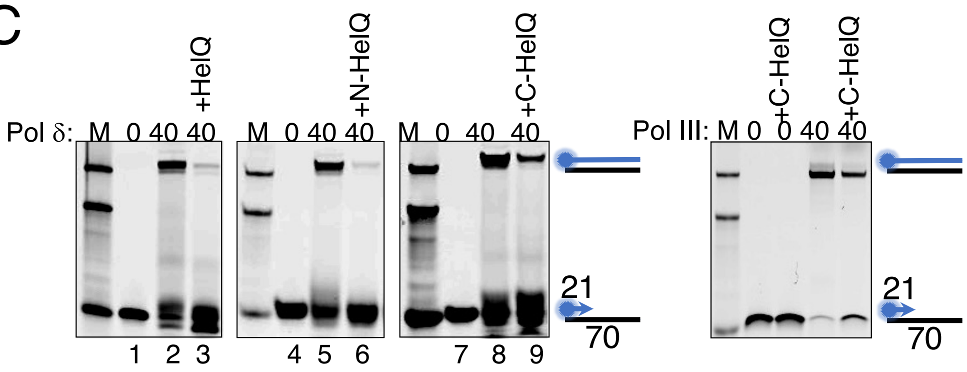

S2D

```
Hs -----MDECGS---------RIRR--RVSLPKRNRPSLGCIFGAPTAAELVPGDEGKEEE   44
Mm -----MEDGCP---------RIRR--RVSVRKRNRGNLENLRASPTPAELQPAEDTEDEA   44
Dr ----------------------------------------MYTDNQLHDIVVKRN-----   15
Ce MNRTPIRRCKSAEIEEDPFSPIPKFSRLRTPRTSR-EYVCPLKSTSPQ--SPSSSTENEP   57
                                                         .

Hs E--MVAE--NRRRKT--AGVLPVE--V-QPLL---LSDSPECLVLGGGDTNPDLLRHMPT   92
Mm A----AG--SRRRKT---------------------------------------------   53
Dr ----TSS--SRKRSR--DGVRS-H--V-TPAKKRSSFTSTTCLADKE----QIYTQDMAE   59
Ce PPVSVTSPPARKRALEESTVTPIQQKIGPPVLKRSSLS-----KLADGFRTAAYLNN---   109
        :    *:*

Hs DRGVGDQPNDSEVDMFGDYDSFT-ENSFIAQVDDLEQKYMQLPEHKKHATDFATENLCSE   151
Mm GSPEHAQENDSEEDMFGDYDSFT-ESSFLAHVDDLEQRYMQLPECGDRDADSGTKDLCSA   112
Dr NEGVEL-CCSDNEDLFEGYDSIVADSSFLAKLEDVELQTRQ---CY----DQQTPNACAD   111
Ce -------ESENDDDPFGL--SFRNEQVLMSK-------------CA-------------P   134
            ..: * *    *:  :. ::::

Hs SIKNKLSITTIGNLTELQTDKHTENQSGYE-GVTIEPGADLLYDVPSSQAIYFENLQNSS   210
Mm GLKNNLRVTTVINLTDPETSEHGQKQSHLDVPAEPEPGSDLSFDVPSSQILYFENPQNSP   172
Dr DLSDSMLAEDFRDSS---PRALPSSQLEFQKA-ITMPHKSPSRGAPSSSTPYLMNPGPAV   167
Ce A-----------------PEKRPET-------LTL----DPSKCLPERDMEMYRKI----   162
                          ..              .     *. .     :

Hs NDLGDHSMKERDWKSS--SHNTVN-----EELPHNCIE----------------------   241
Mm EALGDPCTKKTNGDPQKSSHEELVSSHTEQPEPNNDFSNVRAASESSRRKSLKDHLKSTM   232
Dr NHAD-LSV---------------------------------NKPHPKARRSMKDHLKKVL   193
Ce KK----LDKFYDWQQECLSDKRLL-------DGENCILSLPT------------------   193
   :

Hs --------------------------------------------------   241
Mm AGNARAQT------------------------------------------   240
Dr MDNAATASTVSKMVQQKEAVMNEEMSFAMQAMESIT--SEGDLGPFFGL-   240
Ce ---GAGKTLIAEVLMLREAIVRKRNAILVLPYVAIVQEKISALAPFEDAF   240
```

S3A

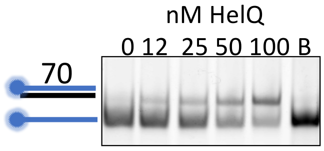
nM HelQ
0 12 25 50 100 B
70

S3B

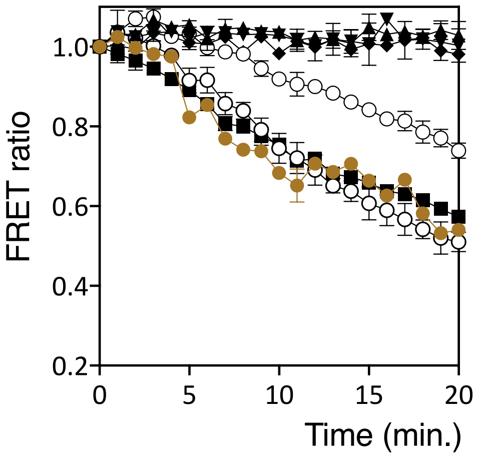
FRET ratio
1.0
0.8
0.6
0.4
0.2
0
5
10
15
20
Time (min.)

S4A

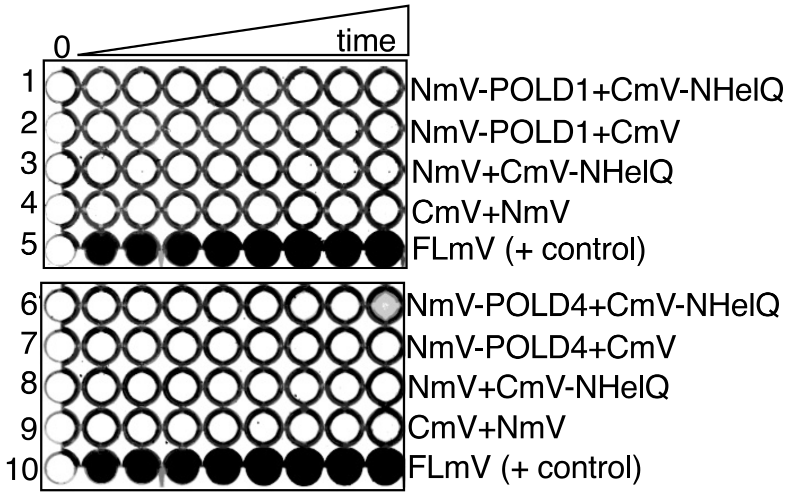
0
time
1
2
3
4
5
6
7
8
9
10
NmV-POLD1+CmV-NHelQ
NmV-POLD1+CmV
NmV+CmV-NHelQ
CmV+NmV
FLmV (+ control)
NmV-POLD4+CmV-NHelQ
NmV-POLD4+CmV
NmV+CmV-NHelQ
CmV+NmV
FLmV (+ control)

S4B i

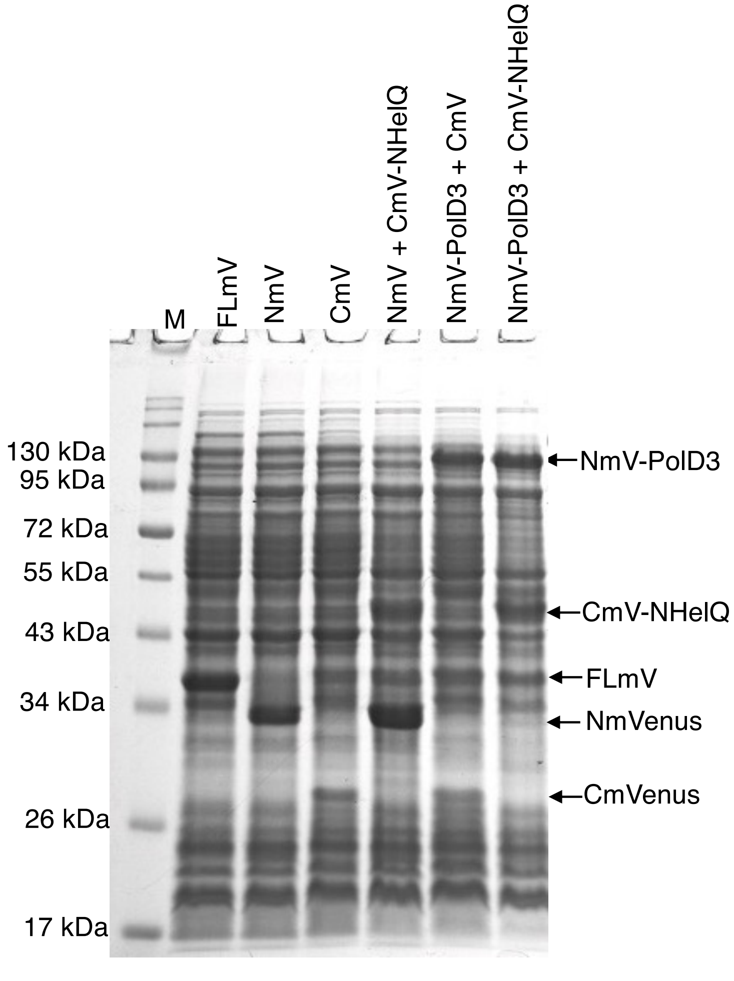
M
FLmV
NmV
CmV
NmV + CmV-NHelQ
NmV-PolD3 + CmV
NmV-PolD3 + CmV-NHelQ
130 kDa
95 kDa
72 kDa
55 kDa
43 kDa
34 kDa
26 kDa
17 kDa
NmV-PolD3
CmV-NHelQ
FLmV
NmVenus
CmVenus

S4B ii

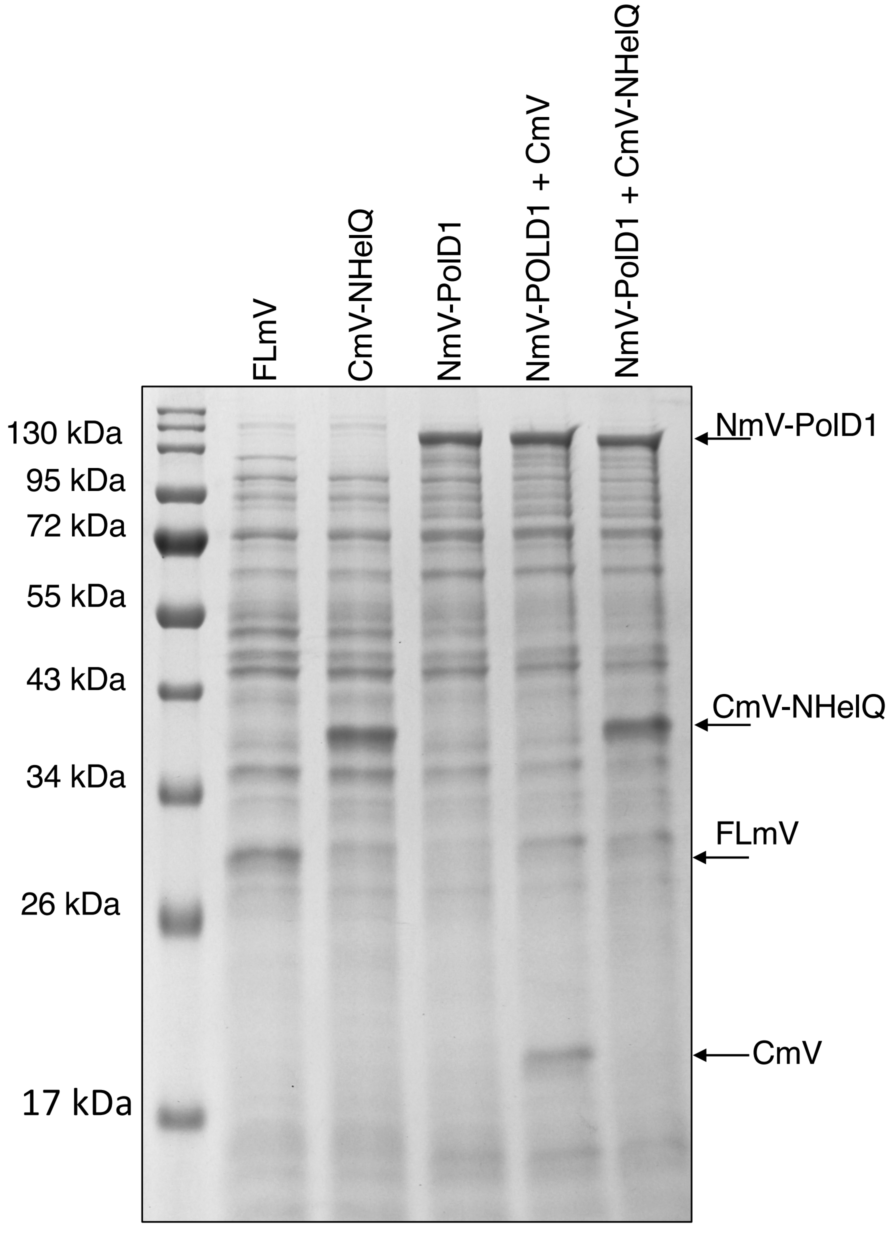
FLmV
CmV-NHelQ
NmV-PolD1
NmV-POLD1 + CmV
NmV-PolD1 + CmV-NHelQ
130 kDa
95 kDa
72 kDa
55 kDa
43 kDa
34 kDa
26 kDa
17 kDa
NmV-PolD1
CmV-NHelQ
FLmV
CmV

S4B iii

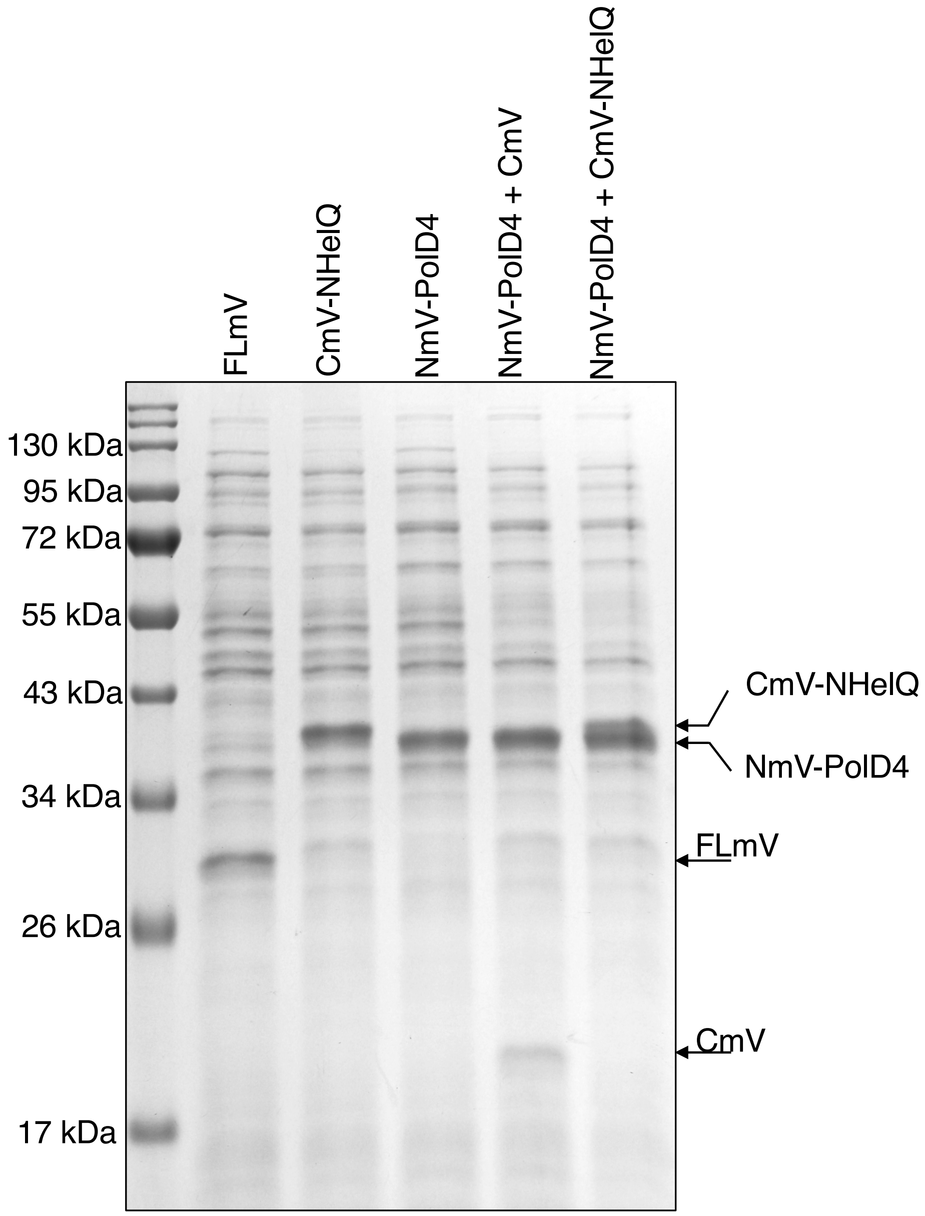

FLmV
CmV-NHelQ
NmV-PolD4
NmV-PolD4 + CmV
NmV-PolD4 + CmV-NHelQ
130 kDa
95 kDa
72 kDa
55 kDa
43 kDa
34 kDa
26 kDa
17 kDa
CmV-NHelQ
NmV-PolD4
FLmV
CmV

S5

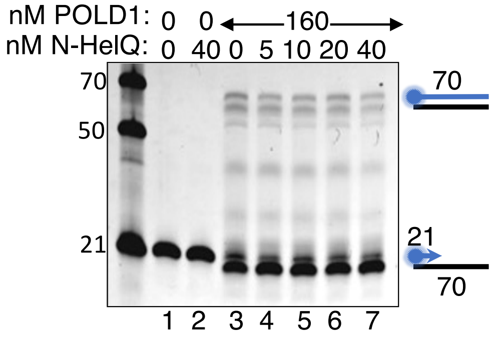

Supplement: gkad032_Supplemental_Files [file gkad032_supplemental_files.zip › R1 Supplementary figures.pdf]
